# Exploring the long‐term effects of COVID‐19 in patients with epilepsy: A multicenter Italian observational study

**DOI:** 10.1002/epi4.70108

**Published:** 2025-08-09

**Authors:** Fedele Dono, Mirella Russo, Giacomo Evangelista, Clarissa Corniello, Claudio Liguori, Carmen Calvello, Flavia Narducci, Catello Vollono, Stefano Sensi

**Affiliations:** ^1^ Department of Neuroscience, Imaging and Clinical Science “G. d'Annunzio” University of Chieti‐Pescara Chieti Italy; ^2^ Behavioral Neurology and Molecular Neurology Units, Center for Advanced Studies and Technology (CAST) and Institute for Advanced Biomedical Technologies (ITAB) University of Chieti‐Pescara Chieti Italy; ^3^ Epilepsy Center, Neurology Institute “SS Annunziata” University Hospital University of Chieti‐Pescara Chieti Italy; ^4^ Department of Systems Medicine University of Rome Tor Vergata Rome Italy; ^5^ Neurology Unit, Epilepsy Centre University Hospital of Rome Tor Vergata Rome Italy; ^6^ Unit of Neurology, Neurophysiology, Neurobiology, Department of Medicine University Campus Bio‐Medico Rome Italy; ^7^ Neurologia, Dipartimento di Neuroscienze, Organi di Senso e Torace Fondazione Policlinico Universitario Agostino Gemelli IRCCS Rome Italy

**Keywords:** epilepsy, healthcare, intervention, post acute sequelae of COVID‐19, SARS‐CoV‐2

## Abstract

**Objective:**

Coronavirus disease‐19 (COVID‐19), caused by SARS‐CoV‐2, has led to a global pandemic since December 2019. People with epilepsy (PwE) face higher risks of severe COVID‐19 outcomes and may be more vulnerable to long‐term neurological and psychiatric effects.

**Methods:**

This multicenter, retrospective cohort study reviewed medical records of PwE with confirmed SARS‐CoV‐2 infection (COVID+) from four Italian hospitals (March 2020–December 2021). A control group (COVID−) included age‐ and sex‐matched PwE without infection. Demographics, epilepsy features, COVID‐19 severity, and neurological/psychiatric outcomes were assessed at baseline and at 6 and 12 months. Statistical analyses included regression and linear mixed model (LMM).

**Results:**

Among 130 patients (38 COVID+, 92 COVID−), no baseline differences were found in demographics, epilepsy characteristics, or comorbidities. At 6 months, the COVID+ group showed increased seizure frequency (*p* = 0.03) and higher rates of psychiatric (*p* < 0.01) and neurological symptoms (*p* < 0.01), requiring specific treatment (*p* = 0.01). At 12 months, psychiatric and neurological disorders persisted (p < 0.01), with more treated neurological symptoms (*p* < 0.01). LMM analysis found no significant seizure frequency differences over time (*p* = 0.47), but focal‐to‐bilateral tonic–clonic seizures showed a time‐dependent interaction (*p* = 0.025).

**Significance:**

SARS‐CoV‐2 infection has lasting neurological and psychiatric effects in PwE. While acute seizure frequency changes are transient, cognitive impairment, insomnia, and depression persist, underscoring the need for continuous monitoring and personalized care.

**Plain Language Summary:**

This study investigated long‐term neurological and psychiatric outcomes in people with epilepsy (PwE) after COVID‐19 infection. We compared 38 PwE with confirmed SARS‐CoV‐2 infection to 92 uninfected controls, matched by age and sex. At 6 months, infected patients showed increased seizure frequency and more psychiatric and neurological symptoms, often requiring treatment. At 12 months, seizure frequency stabilized, but cognitive issues, depression, and insomnia persisted. These findings highlight that while seizure changes may be temporary, COVID‐19 has lasting neuropsychiatric effects in PwE, emphasizing the importance of long‐term monitoring and individualized therapeutic strategies.

**Key points**

- SARS‐CoV‐2 infection causes long‐term neurological and psychiatric symptoms in PwE.
- Seizure frequency increases temporarily post‐infection but stabilizes after 12 months.
- Brain fog and insomnia are common long‐term neurological symptoms in PwE post‐COVID.
- Anxiety and depression are common long‐term psychiatric symptoms in PwE post‐COVID.
- Multidisciplinary care is essential for managing long‐term neurological and psychiatric issues.

## 1 INTRODUCTION

Coronavirus disease 2019 (COVID‐19) is a contagious disease caused by severe acute respiratory syndrome coronavirus 2 (SARS‐CoV‐2). Since the first case was identified in Wuhan, China, in December 2019, the disease has spread worldwide, leading to an ongoing pandemic.^1^ According to the Centers for Disease Control and Prevention (CDC), the risk of SARS‐CoV‐2 infection appears higher in patients suffering from chronic neurological disorders, among which epilepsy is one of the most common, with an estimated prevalence of 0.7%–1%.^2^ In line with this evidence, a recent population‐based study revealed that the incidence of SARS‐CoV‐2 infection, as well as the mortality risk for COVID‐19 in people with epilepsy (PwE), is higher than in the general population.^3^

SARS‐CoV‐2 affects the lower respiratory tract, causing the core clinical symptoms of COVID‐19. However, there is growing evidence that infection with SARS‐CoV‐2 may lead to neurological and psychiatric signs and symptoms which can be evident not only during the acute phase of the infection (i.e., 10 days prior to and 28 days following the SARS‐CoV‐2 infection)^4^ but also after several months. In this regard, the concept of “long‐COVID” or Post Acute Sequelae of COVID‐19 (PASC) has gradually emerged, leading to the concern that survivors of COVID‐19 might be at an increased risk of developing neurological disorders.^5^ These manifestations include milder symptoms such as headaches, hyposmia, hypogeusia, and fatigue to more severe conditions, including sleep disorders, pain, and cognitive impairment.^6^

The pathophysiological mechanisms underlying the onset of neurological and psychiatric long‐COVID symptoms are still unknown, even though chronic neuroinflammation and SARS‐CoV‐2‐induced neuropathological changes (e.g., brain atrophy, and neurovascular damage) seem to play a pivotal role.^7^

Gaining a deeper understanding of the long‐term consequences of COVID‐19 on brain health is essential for developing appropriate diagnostic and therapeutic strategies aimed at improving the long‐term outcomes of COVID‐19 survivors, particularly those with underlying neurological chronic diseases such as epilepsy.^8^

This study aims to identify the neurological and psychiatric long‐term effects of SARS‐CoV‐2 infection in PwE.

## 2 METHODS

### 2.1 Patients selection

In this multicenter, retrospective, observational, cohort study, we reviewed the medical records of patients with diagnosis of epilepsy according to the International League Against Epilepsy (ILAE) criteria and a laboratory‐confirmed SARS‐CoV‐2 infection (COVID+ group) who accessed four third‐level hospitals in the center of Italy (“Policlinico SS Annunziata” of Chieti, “Università Campus Biomedico” of Rome, “Policlinico Universitario Tor Vergata” of Rome, and “IRCCS Policlinico Gemelli” of Rome) between 01/03/2020 and 31/12/2021 (i.e., target period). The specific target period was selected to consider mostly patients infected with SARS‐CoV‐2 alpha (B.1.1.7) and delta (B.1.617.2) strains, thus excluding further variants.

Inclusion criteria were: (1) age over 18 years; (2) SARS‐CoV‐2 infection confirmed by nasopharyngeal swabs or in bronchoalveolar lavage fluid by real‐time polymerase chain reaction (RT‐PCR) analysis, eventually with clinical symptoms reflecting COVID‐19; (3) completion of seizure frequency self‐assessments over the past year; and (4) self‐reported high anti‐seizure medication (ASM) compliance. Exclusion criteria included: (1) poor adherence to ASMs, (2) history of alcohol or drug abuse, (3) diagnosis of psychogenic non‐epileptic seizures (PNES), and (4) history of neurological or psychiatric comorbidities.

A control group of PwE, matched for age and sex, was selected. This group consisted of individuals with a confirmed negative PCR test for SARS‐CoV‐2 infection and no reported symptoms potentially related to COVID‐19 at any visit during the target period as well as during the 12‐month follow‐up (COVID− group). Notably, during the pandemic, outpatient access to the participating public hospitals was permitted only after obtaining a PCR test for SARS‐CoV‐2. Additionally, individuals were instructed—per directives issued by the Italian Ministry of Health—to undergo SARS‐CoV‐2 nasopharyngeal swabs if they experienced probable COVID‐19‐related symptoms or had contact with confirmed SARS‐CoV‐2 cases.^9^ Each patient in the COVID+ group was matched with at least two COVID− controls. Given the absence of reliable data on the expected prevalence of Long‐COVID neurological manifestations in people with epilepsy, a priori sample size estimation was not feasible. The sample size was therefore determined by the number of eligible patients available during the target period.

For both groups, demographics, clinical (e.g., comorbidities), instrumental (e.g., electroencephalogram [EEG]) features and magnetic resonance imaging (MRI) findings and epileptological data (e.g., epilepsy type, etiology and ASM treatment) were collected at baseline (i.e., for COVID+ group: when the patient presented a positive RT‐PCR test for SARS‐CoV‐2; for COVID− group: the first visit available in the time‐period) and at 6‐month and 12‐month follow‐up visits. All patients enrolled at the participating centers completed follow‐up visits at 6 and 12 months, in accordance with standard clinical practice. Of note, seizures’ frequency was assessed by reviewing the medical charts based on the patients' self‐reported diaries. Neurological and psychiatric symptoms at baseline and during the 12‐month follow‐up were collected based on clinical information documented by epileptologists during routine visits. As part of standard clinical practice, these visits include the assessment, classification, and, when necessary, treatment of any emerging neurological or psychiatric symptoms.

Data regarding SARS‐CoV‐2 vaccination (i.e., type of vaccine, date of vaccination, number of doses) were also collected.

The study was performed in agreement with the Helsinki declaration and was approved by the ethics committees of the Catholic University of Rome (ID number: 3076). Informed consent was obtained from all participants.

### 2.2 COVID‐19 severity scale

In the COVID+ group, COVID‐19 severity was classified according to pulmonary symptoms into four categories: asymptomatic, mild, moderate, and severe, based on current clinical guidelines.^10^, ^11^ Specifically: (1) a positive RT‐PCR test for SARS‐CoV‐2 defined asymptomatic COVID‐19 without any clinical signs; (2) mild COVID‐19 referred to cases with a positive RT‐PCR test and upper respiratory symptoms (e.g., pharyngeal congestion, sore throat, and fever) of short duration, or asymptomatic infection, with no abnormal radiographic or septic findings; (3) moderate COVID‐19 involved cases with a positive RT‐PCR test, mild pneumonia, and symptoms such as fever, cough, fatigue, headache, and myalgia; (4) severe COVID‐19 was characterized by a positive RT‐PCR test, moderate or mild clinical features, and signs of disease progression, including respiratory failure requiring mechanical ventilation (e.g., acute respiratory distress syndrome, persistent hypoxia unresponsive to supplemental oxygen), septic shock, and/or organ failure requiring intensive care unit monitoring.

### 2.3 Categorization of long‐term neurological symptoms in COVID‐19+ patients

Neurological symptoms were associated with long COVID‐19 if they appeared at least 4 weeks after a positive SARS‐CoV‐2 PCR test or the onset of COVID‐19 symptoms. Previous studies^12^ categorized neurological signs into four groups: no neurological symptoms, nonspecific, mild, and severe neurological symptoms. Specifically: (1) No neurological symptoms referred to the absence of any symptoms, as determined by clinical examination and patient history; (2) nonspecific neurological symptoms included mild symptoms (e.g., dizziness, headache, muscle pain, general weakness, brain fog) that emerged more than 30 days after a positive SARS‐CoV‐2 PCR test or the onset of COVID‐19 symptoms; (3) Mild neurological symptoms encompassed mild symptoms such as altered taste or smell, cranial nerve paresis, or sleep disturbances, with onset following the same timeline; and (4) Severe neurological symptoms included more serious symptoms, such as acute cerebrovascular events, severe sleep disorders, movement disorders, myopathy, or neuropathy, developing more than 30 days after infection or the onset of acute symptoms. The duration of neurological symptoms and the need for a specific treatment were assessed throughout a 12‐month follow‐up period.

### 2.4 Categorization of long‐term psychiatric symptoms in COVID‐19+ patients

Psychiatric symptoms were considered long‐term COVID‐19 sequelae if they had an onset 4 weeks after a positive SARS‐CoV‐2 PCR test or after the onset of COVID‐19 symptoms. Psychiatric symptoms were reviewed and classified according to the Diagnostic and Statistical Manual of Mental Disorders Fifth Edition (DSM‐5). The duration of psychiatric symptoms and the need for a specific treatment were assessed throughout a 12‐month follow‐up period. Psychiatric symptoms that did not require any pharmacological treatment were categorized as mild in contrast to those deserving an established treatment defined as moderate. Consequently, severe psychiatric symptoms were considered those for which hospitalization was required.

### 2.5 Endpoints

The primary endpoint was the change in mean monthly seizure frequency at the 6‐ and 12‐month follow‐up visits, compared to the mean monthly seizure frequency during the 12 months before enrollment. To that aim, only objectively countable seizures (i.e., focal aware seizure with motor signs, focal unaware seizure with or without motor signs, focal‐to‐bilateral tonic–clonic seizures—FBTCS, as well as generalized motor and non‐motor seizures) were considered. Secondary outcomes included the prevalence of long‐term neurological and psychiatric symptoms and any changes in the ASM regimen at 6‐ and 12‐month follow‐up visits.

### 2.6 Statistical analysis

We compared baseline features between COVID+ and COVID− participants to detect significant differences. Normal distribution was assessed using the Shapiro–Wilk test. For parametric data, means (standard deviations, SD) would be applied; for nonparametric data, medians (interquartile ranges, IQR) were reported; percentages were used for categorical data. Chi‐square analysis was performed to compare categorical variables, whereas the Mann–Whitney U test was applied to compare quantitative data. Subsequently, regression analyses were conducted. First, univariate regression assessed variables of interest concerning outcomes, reporting odds ratios (ORs) and *p*‐values. Collinearity among selected variables (*p* < 0.20) was evaluated through correlation analysis. If Pearson's *r* exceeded 0.90 with a significant *p*‐value (<0.05), one variable from each pair was excluded based on clinical and scientific rationale. The remaining variables were then used for multivariate regression analyses: logistic regression for categorical variables and linear regression for continuous data. Model fit was assessed using deviance, Akaike's information criterion (AIC), and McFadden's *R* ^2^, along with the variance inflation factor (VIF) for included variables, allowing tolerance for VIF values <10. The significance level was set at *p* < 0.05. Finally, a Linear Mixed Model analysis was conducted to evaluate the effect of time on longitudinal assessments. Statistical analyses were performed using Jamovi software (version 2.3.24).

## 3 RESULTS

### 3.1 Demographics and clinical features of the COVID+ group

Thirty‐eight patients (16 males, median age 40.8 y.o., IQR 18.4) with a PCR‐based SARS‐CoV‐2 infection were included in the study (see Table 1 for cohort features). All included subjects showed an intermediate‐high level of education (mean years of education ± SD: 11.9 ± 4.19), and 52.6% had stable employment. More than half the subjects (57.8%) exhibited comorbidities, mostly cardiovascular diseases (i.e., hypertension and tachycardia), dyslipidemia, diabetes, and inflammatory joint disease.

**TABLE 1:** Demographic characteristics and clinical features.

	COVID+ group (*n* = 38)	COVID− group (*n* = 92)	*p*‐Value
**Demographics**			
Age (mean ± SD)	40.8 ± 18.4	36 ± 19.5	0.66
Sex (M)	16	38	0.93
Education (mean ± SD)	11.9 ± 4.19	11.1 ± 3.27	0.34
Employment	20	28	0.06
Comorbidities	22	52	0.89
**Epilepsy features**			
Seizure type			
Focal	35	87	0.97
Focal‐to‐bilateral	23	68	0.11
Generalized tonic‐clonic	3	5	0.59
Etiology			
Structural	8	23	0.63
Unknown	30	69	0.63
Disease duration (mean ± SD)	15 ± 13.8	17.3 ± 16.6	0.83
Seizure frequency (12 months before)	0.08 (0.33)	0.08 (0–1)	0.69
FBTCS frequency (12 months before)	0 (0)	0 (0.08)	0.13
ER access (12 months before)	3	13	0.32
Previous status epilepticus	1	2	0.88
ASM at baseline (IQR)	1 (1)	1 (1)	0.95
Levetiracetam	11	27	
Lamotrigine	5	13	
Lacosamide	4	6	
Carbamazepine	9	22	
Valproate	5	20	
Phenobarbital	2	6	
Topiramate	3	5	
Perampanel	1	6	
Brivaracetam	1	4	
Drug‐resistant epilepsy	2	9	0.40
**COVID‐19**			
COVID‐19 scale	1 (0)	0 (0)	
1	5	0	
2	29	0	
3	3	0	
4	1	0	
COVID‐19 vaccine	23	77	<0.01

Focal epilepsy was the most prevalent diagnosis (92.1%), whereas generalized epilepsy with tonic–clonic seizures was reported in 7.9% of subjects. Patients showed a median epilepsy history of 15 (IQR 13.8) years, mostly associated with an unknown underlying etiology (78.9%). The median number of administered ASM was 1 (IQR 1). Epilepsy was generally well controlled, except for two individuals (5.26%) suffering from drug‐resistant epilepsy (DRE). The median monthly seizure frequency, calculated during the 12 months prior to the study enrollment, was 0.08 (IQR 0.33), with a median tonic–clonic monthly seizure frequency of 0 (IQR 0). Only 3 patients (7.9%) reported emergency room (ER) access due to seizure recurrence in the previous year, whereas just one patient presented with status epilepticus in the previous 12 months.

According to the COVID‐19 severity scale, 5 patients (13.1%) showed an asymptomatic SARS‐CoV‐2 infection (score: 1), 29 (76.3%) a mild infection (score: 2), whereas moderate and severe clinical pictures were reported in 3 (7.9%) (score: 3) and 1 (2.6%) (score: 4) case, respectively. At baseline, 23 patients (60.5%) had already received at least one dose of SARS‐CoV‐2 vaccination (median: 2, IQR 1), and the Pfizer‐BioNTech COVID‐19 Vaccine was the most administered.

At 6 months, 10 patients showed an increased monthly seizure frequency compared to baseline. In addition, 22 patients complained of long‐term psychiatric symptoms, among which anxiety and depression were mostly reported. Furthermore, 23 patients also indicated long‐term neurological manifestations, which generally consisted of brain fog, cephalalgia, and sleep disorders (i.e., insomnia). At 6 months, long‐term psychiatric symptoms were classified as mild in almost all cases, requiring a specific treatment in 5. Neurological symptoms were classified as non‐specific in all but three cases, which were classified as severe (i.e., 2 patients with parkinsonism and 1 patient with cognitive impairment) and needing specific treatment.

At 12 months, 17 patients showed an increased monthly seizure frequency compared to the 12‐month period prior to the enrollment. Moreover, 22 patients still complained of long‐term psychiatric symptoms, whereas 21 complained of long‐term neurological ones. Long‐term psychiatric symptoms were classified as mild in 18 cases, whereas 4 patients required specific treatments. Long‐term neurological symptoms were classified as non‐specific in all cases but 2 cases (i.e., cognitive impairment), with 6 patients requiring a specific treatment.

### 3.2 Demographics and clinical features of the COVID− group

Ninety‐two patients (38 males, median age: 36, IQR 19.5 years) with no history of SARS‐CoV‐2 infection, as confirmed by negative nasal swabs and no COVID‐19 symptoms, were selected. All included patients showed an intermediate‐high level of education (median years of education: 11.1, IQR 3.27), and 71.8% reported stable employment. Fifty‐two patients (56.5%) exhibited comorbidities, mostly cardiovascular disease (i.e., hypertension and tachycardia), dyslipidemia, diabetes, and inflammatory joint disease.

Also, in this group, the diagnosis of focal epilepsy was largely prevalent (94.9%), whereas generalized epilepsy with tonic–clonic seizures was reported in only 5.4% of cases. Patients showed a median epilepsy duration of 17.3 years (IQR 16.6), primarily due to an unknown underlying etiology (82%). The median number of administered ASM was 1 (IQR 1). Nine patients (9.8%) were diagnosed with DRE. The median overall monthly seizure frequency calculated during the 12 months before the study enrollment was 0.08 (IQR 1), with a median tonic–clonic monthly seizure frequency of 0 (IQR 0.08). Thirteen patients (10.2%) reported ER access due to seizure recurrence in the previous year, whereas 2 patients had suffered from a SE in the previous 12 months.

At baseline, 77 patients (69.2%) had already received at least one dose of SARS‐CoV‐2 vaccination (median: 2, IQR 1), with the Pfizer‐BioNTech COVID‐19 Vaccine being the most administered.

At 6 months, 10 patients showed an increased monthly seizure frequency compared to baseline. Fourteen patients complained of long‐term psychiatric symptoms, among which irritability and depression were mostly reported. Furthermore, 13 patients referred long‐term neurological manifestations, which generally consisted of brain fog and sleep disorders (i.e., insomnia). At 6 months, long‐term psychiatric symptoms were classified as mild‐to‐moderate in all cases, whereas neurological symptoms were classified as non‐specific in all but 1 case, which was classified as severe (i.e., 1 patient with parkinsonism).

At 12 months, 9 patients showed an increased monthly seizure frequency compared to the 12‐month period prior to enrollment. Fourteen patients still complained of long‐term psychiatric symptoms, whereas 16 had long‐term neurological ones. Long‐term psychiatric symptoms were classified as mild‐to‐moderate in all cases but required a specific treatment in 10. Long‐term neurological symptoms were classified as non‐specific in all cases but 2 (i.e., cognitive impairment and parkinsonism), with only 1 patient requiring a specific treatment.

### 3.3 Comparison between groups

At baseline, no differences were detected between COVID− and COVID+ groups (Table 1) according to the level of education, employment, epilepsy characteristics, number of ASM administered, as well as comorbidity. However, patients in the COVID+ group showed a significantly lower rate of SARS‐CoV‐2 vaccination (*p* < 0.01).

At 6 months (Table 2), COVID+ subjects reported increased monthly seizure frequency more often than the COVID− group (*p* = 0.03). No differences were detected according to ER access due to epileptological emergencies or ASM numbers. In addition, patients in the COVID+ group showed a higher prevalence of long‐term psychiatric symptoms (*p* < 0.01) as well as long‐term neurological ones (*p* < 0.01). Long‐term psychiatric symptoms required a specific treatment in 5 cases in the COVID+ group and 14 in the COVID‐ group (*p* = 0.74). In contrast, only in the COVID+ group, the new neurological symptoms required specific treatment (*p* = 0.01).

**TABLE 2:** Six‐month and 12‐month follow‐up characteristics of COVID+ and COVID− groups.

	COVID+ group	COVID− group	*p*‐value
	(*n* = 38)	(*n* = 92)	
6 months follow‐up			
Increased mean monthly seizure frequency	10	10	0.03
ASM number (IQR)	1 (1)	1 (1)	0.61
Levetiracetam	9	31	
Lamotrigine	8	16	
Lacosamide	5	4	
Carbamazepine	2	20	
Valproate	6	20	
Phenobarbital	2	4	
Topiramate	2	7	
Perampanel	4	8	
Brivaracetam	1	5	
ER access due to epileptological emergencies	3	2	0.13
Long‐term psychiatric symptoms	22	14	<0.01
Depression	8	6	
Anxiety	10	3	
Irritability	4	5	
Panic attack	1	0	
Psychiatric new treatments	5	14	0.74
Long‐term neurological symptoms	23	13	<0.01
Brain fog	9	5	
Headache	6	3	
Insomnia	5	4	
Tremor	2	1	
Cognitive impairment	1	0	
Neurological new treatments	3	0	0.01
12 months follow‐up			
Increased mean monthly seizure frequency	17	9	0.50
ASM number (IQR)	1 (1)	1 (1)	0.51
Levetiracetam	9	28	
Lamotrigine	7	15	
Lacosamide	6	4	
Carbamazepine	3	20	
Valproate	5	18	
Phenobarbital	2	4	
Topiramate	1	7	
Perampanel	4	9	
Brivaracetam	1	6	
ER access due to epileptological emergencies	1	1	0.52
Long‐term psychiatric symptoms	22	14	<0.01
Depression	6	5	
Anxiety	10	3	
Irritability	5	5	
Panic attack	1	1	
Psychiatric new treatments	4	10	0.95
Long‐term neurological symptoms	21	16	<0.01
Brain fog	6	6	
Headache	8	5	
Insomnia	5	3	
Tremor	0	1	
Cognitive impairment	2	1	
Neurological new treatments	6	1	<0.01

Abbreviation: ASM, anti‐seizure medication.

At 12 months (Table 2), no differences were observed between the two groups in terms of mean monthly seizure frequency variation, ER access due to epileptological emergencies, or ASM regimen. However, patients in the COVID+ group still showed a higher prevalence of long‐term psychiatric disorders (*p* < 0.01) and neurological disorders (*p* < 0.01). Even at 12 months, the number of individuals who required a specific treatment for long‐term neurological symptoms was higher in the COVID+ group (*p* < 0.01).

### 3.4 Regression analyses

A multivariate stepwise logistic regression was carried out to assess the role of SARS‐CoV‐2 infection concerning the onset of long‐term psychiatric or neurological symptoms and mean monthly seizure frequency changes at 6‐ and 12‐month follow‐up. At the univariate regression, SARS‐CoV‐2 infection was significantly associated with mean monthly seizure frequency change at 6‐month (*p* = 0.03, OR = 2.92, 95% IC = 1.10–7.77), and the onset of long‐term psychiatric symptoms at the two follow‐ups (6‐month: *p* < 0.001, OR 7.56, 95% IC = 3.20–17.87, 12‐month: *p* < 0.001, OR = 7.56, 95% IC = 3.20–17.87) and long‐term neurological symptoms as well (6‐month: *p* < 0.001, OR = 9.20, 95% IC = 3.83–22.10, 12‐month: *p* < 0.001, OR = 5.87, 95% IC = 2.54–13.54).

**FIGURE 1:**
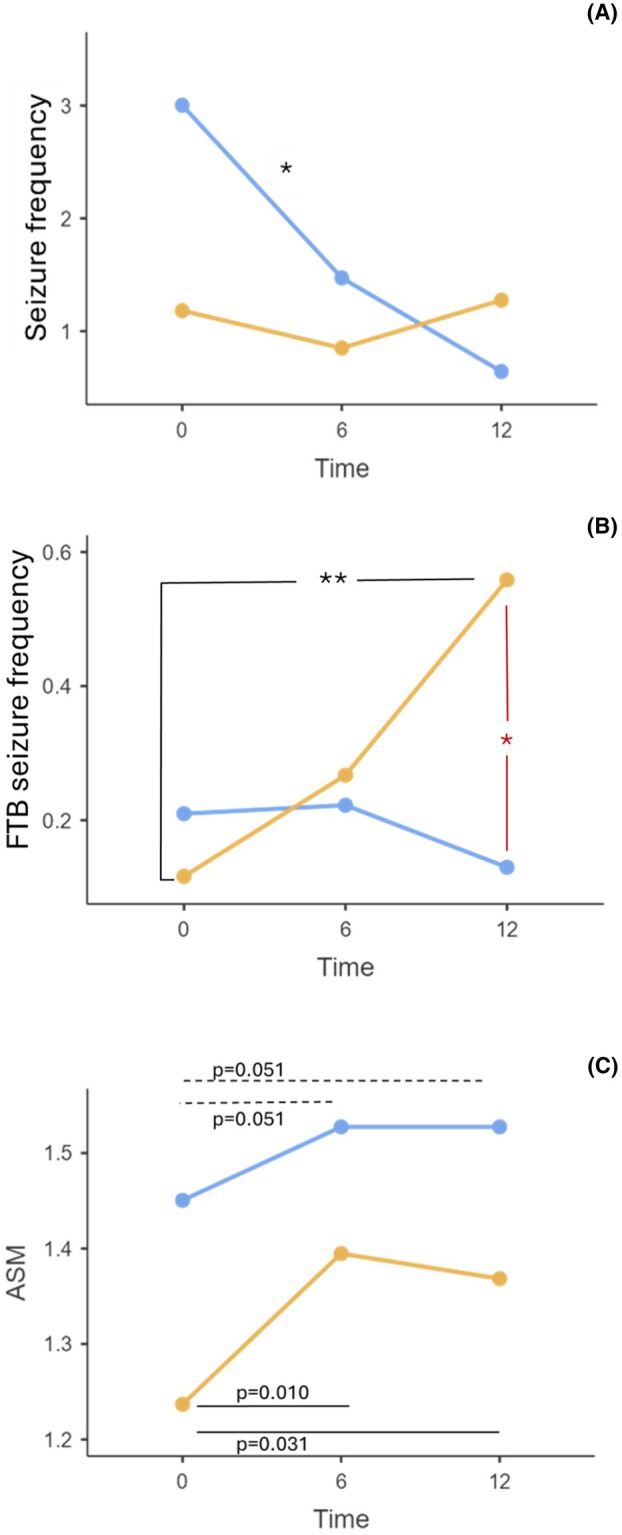
LMM analysis. Blue and yellow lines refer to COVID− group and COVID+ group respectively. (A) LMM for seizure frequency changes over time in COVID+ and COVID− subjects. The figure shows the changes in seizure frequency over time in the two groups. No statistically significant differences were found, except for the changes in seizure frequency in the control group over time (in‐group, time effect at the diagnosis × time post‐hoc comparisons, *T0–T6, *p* = 0.03). (B) LMM for FBTCS frequency changes over time in COVID+ and COVID− subjects. The figure shows the changes in FBTCS frequency over time in the two groups. All differences were not significant, except for the changes in seizure frequency in the COVID group over time (in‐group, time effect at the diagnosis × time post‐hoc comparisons, **T0–T12, *p* = 0.008). Moreover, a significant difference in FBTCS frequency was observed at T12 between the groups (between‐groups, diagnosis effect at the diagnosis × time post‐hoc comparisons, *T12 *p* = 0.022). (C) LMM for ASM number changes over time in COVID+ and COVID−subjects. The figure shows the changes in ASM number over time in the two groups. All differences between groups were not significant. However, an impact of time was observed in both groups at the post‐hoc analysis (trend in controls, statistically significant in COVID+ subjects). In controls, the increase was observed at T6 and appeared “stabilized” at T12. In COVID+ subjects, a marked increase in ASM was observed at T6 compared to baseline, with a mild decline at T12. ASM, anti‐seizure medication; FBTCS, focal‐to‐bilateral tonic–clonic seizures; LLM, linear mixed model.

At the multivariate regression, SARS‐CoV‐2 infection retained a statistical significance as a predictor of the onset of long‐term psychiatric and neurological symptoms only at 6 months (respectively, *p* < 0.001, OR = 9.63, 95% IC = 3.72–24.96 and *p* < 0.001, OR = 12.11, 95% IC = 4.10–35.77, see Tables 3 and 4 for complete details on the models). Interestingly, access to ER within the previous 12 months was a significant predictor of long‐term psychiatric and neurological symptoms at 6 months. Finally, comorbidities significantly increased the risk of long‐term neurological symptoms within the first 6 months of follow‐up. At 12 months, the presence of 6‐month long‐term psychiatric symptoms remained a statistically significant predictor of long‐term neurological symptoms at 12 months (OR = 6.80, 95% IC = 2.26–20.49, *p* < 0.001). Furthermore, the presence of long‐term psychiatric and neurological symptoms at 6 months also served as a predictor of the onset of long‐term psychiatric symptoms only at 12 months (respectively, *p* < 0.001, OR = 22.13, 95% IC = 6.57–74.48 and *p* = 0.018, OR = 4.61, 95% IC = 1.30–16.38, see Tables S1 and S2 for complete details on the models). Furthermore, a linear mixed model (LMM) analysis was applied to assess the interaction between SARS‐CoV‐2 infection and time in relation to the overall frequency of seizures, the frequency of FBTCS seizures, and the number of ASM administered. Although a different trend was observed between COVID+ subjects and controls (Figure 1A), no significant difference was observed between the two groups according to seizure frequency (*p* = 0.47) except for the changes in seizure frequency in the COVID− group at 6 months (T0–T6, *p* = 0.03). The changes in FBTCS seizure frequency were not significantly different between groups (0 = 0.39) except when considering the interaction with time (diagnosis × time, *p* = 0.025; time effect was not significant alone with *p* = 0.188). At the 12‐month follow‐up, a significant difference was observed between the two groups in the post‐hoc analysis (Figure 1B).

**TABLE 3:** Multivariate logistic regression for the outcome of “long‐term neurological symptoms” at 6 months.

Predictor	Estimate	SE	*Z*	*p*	Odds ratio	95% confidence interval	
						Lower	Upper
Intercept	−3.542	1.3564	−2.61	0.009	0.0289	0.00203	0.413
Diagnosis: COVID+/COVID−	2.494	0.5527	4.512	<0.001	12.1075	4.09794	35.772
Education	0.115	0.0781	1.468	0.142	1.1215	0.96228	1.307
FBTCS 12 M before	−3.628	2.3296	−1.55	0.119	0.0266	2.76e‐4	2.556
ER access 12 M before SI	2.120	0.8130	2.608	0.009	8.3309	1.69317	40.991
Comorbidities	1.197	0.5640	2.123	0.034	3.3114	1.09631	10.002
ASM regimen at baseline	−0.281	0.4067	−0.69	0.490	0.7551	0.34028	1.676

*Note*: Estimates represent the log odds of “Long‐term neurological symptoms 6‐month = Yes” versus “Long‐term neurological symptoms 6‐month = No.”

Abbreviations: ASM, anti‐seizure medications; ER, emergency room; FBTCS, focal‐to‐bilateral tonic–clonic seizures; SI, study inclusion.

**TABLE 4:** Multivariate logistic regression for the outcome “Long‐term psychiatric symptoms” at 6‐month.

Predictor	Estimate	SE	*Z*	*p*	Odds ratio	95% confidence interval	
						Lower	Upper
Intercept	−2.1041	0.4385	−4.79	<0.001	0.122	0.0516	0.288
ER access 12M Before SI	1.4052	0.6501	2.162	0.031	4.076	1.1400	14.575
Diagnosis: COVID+/COVID−	2.2651	0.4858	4.662	<0.001	9.632	3.7168	24.961
Previous SE	1.5567	1.3680	1.138	0.255	4.743	0.3248	69.270
Seizure frequency 12M before SI	−0.0602	0.0586	−1.02	0.305	0.942	0.8394	1.056
Sex: M–F	0.2365	0.4658	0.508	0.612	1.267	0.5084	3.157

*Note*: Estimates represent the log odds of “Long‐term psychiatric symptoms 6‐month = Yes” versus “Long‐term psychiatric symptoms 6‐month = No”.

Abbreviations: ER, emergency room; SE, status epilepticus; SI, study inclusion.

Finally, the LMM was employed to assess differences between the two groups in terms of ASM number over time. Although the diagnosis of SARS‐CoV‐2 infection did not significantly impact (*p* = 0.271), a time effect was found (T0–T6, *p* = 0.01; T0–T12, *p* = 0.031), which was not retained when considering the interaction (diagnosis × time, *p* = 0.521) (Figure 1C).

## 4 DISCUSSION

Five years after the onset of the COVID‐19 pandemic, the long‐term consequences of SARS‐CoV‐2 infection continue to pose a significant public health challenge, affecting millions globally.^13^ In the early stages of the pandemic, insufficient recognition of persistent symptoms complicated the diagnosis and management of long COVID, often resulting in delayed diagnoses and instances of medical neglect. Although the clinical features of long‐term COVID‐19 have been extensively studied in recent years, a comprehensive understanding of its impact on PwE remains lacking.

Our study evaluated the impact of SARS‐CoV‐2 infection on seizure frequency over time, focusing on its transient and long‐term effects in PwE. The findings indicate that individuals with epilepsy who contracted SARS‐CoV‐2 exhibited an increase in seizure frequency at the 6‐month follow‐up compared to those without the infection, even though this difference was no longer evident at 12 months. However, multivariate analysis demonstrated that SARS‐CoV‐2 infection was not an independent predictor of increased seizure frequency for all seizure types at either 6 or 12 months. Accordingly, LMM analysis did not confirm an interaction between group and time when considering seizure frequency changes. Nevertheless, in the subgroup of patients with FBTCS, LMM analysis revealed a significant interaction between time and SARS‐CoV‐2 infection at the 12‐month follow‐up. These findings suggest that transient factors associated with the acute phase of infection may contribute to a temporary exacerbation of seizures. In contrast, the long‐term course of seizure frequency appears unaffected by the infection.^14^, ^15^ Nonetheless, concerns remain for patients with FBTCS, whose frequency may persistently increase even 12 months after the viral infection.

As a secondary outcome, we assessed the onset of long‐term psychiatric and neurological symptoms at 6‐ and 12‐month follow‐up following SARS‐CoV‐2 infection. Our analysis showed that patients with a history of SARS‐CoV‐2 infection were more likely to develop these symptoms than those without infection. In line with existing literature, brain fog and insomnia emerged as the most common neurological symptoms associated with long‐term COVID‐19, even in PwE, often necessitating targeted interventions. Similarly, anxiety and depression were identified as the most frequent psychiatric symptoms. These symptoms persisted at the 12‐month follow‐up, underscoring the prolonged impact of the infection on neurological and psychiatric well‐being. Multivariate analysis confirmed that SARS‐CoV‐2 infection was a significant predictor of long‐term neurological and psychiatric symptoms. Furthermore, our findings indicate that patients with a higher number of ER accesses in the 12 months preceding the infection as well as those with a higher burden of comorbidities were more likely to develop these symptoms, suggesting greater vulnerability among individuals with a more severe clinical picture. Notably, this association was independent of the severity of the initial infection, aligning with previous reports in the literature.^16^, ^17^ Furthermore, the onset of these symptoms was not associated with the specific ASM therapy administered at baseline as well as during the different follow‐ups.

The mechanisms underlying the long‐term neurological and psychiatric symptoms associated with SARS‐CoV‐2 infection have been extensively discussed in the literature. Evidence indicates that patients recovering from COVID‐19 may experience long‐term neuropathological sequelae, such as hippocampal and cortical atrophy, hypoxic–ischemic injury, and small vessel disease. These changes may result from the direct effects of SARS‐CoV‐2 on the CNS and secondary mechanisms involving neuroinflammation and oxidative stress induced by systemic viral infection.^18^

The neuroinvasive, neurotropic, and neurovirulent properties of SARS‐CoV‐2 have been well documented in the literature.^19^ Recent findings suggest that SARS‐CoV‐2 spike proteins can persist beyond the acute phase of infection in specific CNS structures, including the meninges and brain parenchyma.^20^ This persistence may lead to sustained immune activation, contributing to vascular and parenchymal damage. Additionally, systemic viral effects play a significant role. For instance, recent studies have shown that patients with long‐COVID symptoms exhibit elevated serum levels of tumor necrosis factor‐α and interferon‐*γ*–induced protein 10, with a trend toward increased interleukin‐6 levels.^21^ Peripherally released cytokines can influence the brain through two mechanisms, with the first involving a neural pathway (wherein primary afferent neurons, such as the vagus nerve, transmit signals originating from the site of infection), and the second operating via a humoral route (whereby peripheral cytokines cross the blood–brain barrier thereby stimulating brain immune cells to produce proinflammatory cytokines). Furthermore, cytokines are critical in modulating the production, release, and reuptake of key central neurotransmitters, including noradrenaline, dopamine, glutamate, serotonin, GABA, and acetylcholine.^22^ Dysregulation of these processes can disrupt normal neurotransmission, contributing to the development of neurological and psychiatric symptoms related to long‐COVID, including seizure exacerbation.^23^

In terms of specific interventions for PwE and long COVID, our findings suggest that while seizure frequency may require close monitoring, especially during the first 6 months following infection, neurological and psychiatric symptoms warrant a longer follow‐up. Interventions may vary depending on the type of symptoms.^24^ For instance, patients showing neurological symptoms, such as cognitive dysfunction and insomnia, may benefit from targeted assessments, which include neuropsychological testing, sleep questionnaires evaluating the quality of the sleep, or the presence of daytime sleepiness up to polysomnography in selected cases. These assessments could be valuable in monitoring changes and allow for timely interventions, such as cognitive rehabilitation or pharmacological treatments to improve sleep quality and cognitive performance. For psychiatric symptoms, regular psychiatric evaluations should be conducted to assess the need for therapy, medication, or other non‐pharmacological interventions.^25^

Moreover, it is important to recognize that certain ASMs may exacerbate neurological and psychiatric conditions, including impairments in sleep quality, cognitive functioning, and the severity of depressive symptoms.^26^, ^27^ Consequently, the diagnosis of psychiatric or neurological manifestations associated with long COVID may warrant revision of the ASM regimen.

Overall, these interventions should be individualized and based on regular, structured evaluations, considering the neurological and psychiatric aspects of recovery. A multidisciplinary approach involving neurologists, psychiatrists, psychologists, and other healthcare professionals is essential to provide comprehensive care for these patients, ensuring timely management of symptoms and improving long‐term outcomes.

### 4.1 Limitations

Several limitations should be acknowledged in this study. A major limitation is the lack of a priori power calculation, as no previous data were available to estimate the expected prevalence of Long‐COVID symptoms in patients with epilepsy. Furthermore, as an observational retrospective study, causal relationships cannot be firmly established. In addition, some information related to the type and characteristics of neurological and psychiatric symptoms may not have been consistently reported in the clinical records. Moreover, the absence of standardized assessments to objectively evaluate the variety and severity of these symptoms represents a limitation, as they were derived from clinical judgment during standard care visits. Seizure frequency and psychiatric symptoms were assessed through patient self‐reporting, which may have introduced recall or reporting bias. Although several confounders were considered in the analysis, it is possible that unmeasured factors, such as socio‐economic status and genetic predispositions, may have influenced the outcomes. Additionally, we acknowledge that ASM may contribute to neurological and psychiatric adverse events. However, both groups in our study were comparable in terms of the number and types of ASMs used, minimizing the likelihood that treatment differences accounted for the observed outcomes. Furthermore, the relatively small size of the COVID+ group (*n* = 38) compared to the control group (*n* = 92) could have limited the statistical power, particularly for subgroup analyses. The 12‐month follow‐up period might also be insufficient to fully capture long‐term neurological and psychiatric consequences of COVID‐19. Lastly, the large number of statistical tests performed increases the risk of spurious associations that may not reflect true underlying relationships.

## 5 CONCLUSION

This multicenter retrospective study indicates that SARS‐CoV‐2 infection in PwE is associated with transient increases in seizure frequency and persistent long‐term neurological and psychiatric sequelae. Including data from four Italian tertiary hospitals enhances the generalizability of the findings. Detailed classification of neurological and psychiatric symptoms facilitates a deeper understanding of long‐term sequelae. Although the acute exacerbation of seizures observed at 6 months appears to be resolved by 12 months, the sustained prevalence of symptoms such as cognitive impairment, insomnia, and depressive features highlights the prolonged impact on the neuropsychiatric health of PwE. These findings emphasize the necessity for continued longitudinal surveillance and the implementation of individualized, multidisciplinary care strategies. Future studies should employ prospective designs with larger cohorts and extended follow‐up periods to further elucidate the mechanistic underpinnings and optimal management approaches for long‐term manifestations of COVID‐19 in the epilepsy population.

## AUTHOR CONTRIBUTIONS

Fedele Dono and Giacomo Evangelista conceived the study and drafted the original manuscript. Mirella Russo performed the statistical analysis. Clarissa Corniello contributed to data collection. All authors contributed to critical revision and approved the final version of the manuscript.

## CONFLICT OF INTEREST STATEMENT

The authors declare no conflicts of interest related to the present study. We confirm that we have read the Journal's position on issues involved in ethical publication and affirm that this report is consistent with those guidelines.

## Supporting information


Table S1

Table S2

## Data Availability

The data supporting the findings of this study are available upon request from the corresponding author. Due to privacy and ethical restrictions, the data are not publicly available. The study complied with all relevant ethical guidelines, and participant consent was obtained for the use of personal and clinical data.
