# Supplementary material for: Exploring the long‐term effects of COVID‐19 in patients with epilepsy: A multicenter Italian observational study
Source: Epilepsia Open. 2025 Aug 9;10(5):1450–61. doi: 10.1002/epi4.70108 (PMC12514380; doi:10.1002/epi4.70108)
Supplement: Supplementary file 1 — Table S1 [file EPI4-10-1450-s001.docx]

|  | | | | | | | | | | | | | | | |  |
| --- | --- | --- | --- | --- | --- | --- | --- | --- | --- | --- | --- | --- | --- | --- | --- | --- |
|  | | | | | | | | | | | | | **95% Confidence Interval** | | |  |
| **Predictor** | | **Estimate** | | **SE** | | **Z** | | **p** | | | **Odds ratio** | | **Lower** | | **Upper** |  |
| Intercept |  | -1.300 |  | 0.662 |  | -1.964 |  | | 0.050 |  | 0.272 |  | 0.0744 |  | 0.997 |  |
| Diagnosis: |  |  |  |  |  |  |  | |  |  |  |  |  |  |  |  |
| Post Covid – Control |  | 0.974 |  | 0.953 |  | 1.022 |  | | 0.307 |  | 2.650 |  | 0.4089 |  | 17.172 |  |
| DRE: |  |  |  |  |  |  |  | |  |  |  |  |  |  |  |  |
| Yes – No |  | 0.788 |  | 1.501 |  | 0.525 |  | | 0.600 |  | 2.200 |  | 0.1160 |  | 41.729 |  |
| COVID Scale |  | -0.374 |  | 0.762 |  | -0.491 |  | | 0.623 |  | 0.688 |  | 0.1546 |  | 3.060 |  |
| FTB frequency 6m |  | 0.437 |  | 0.257 |  | 1.700 |  | | 0.089 |  | 1.548 |  | 0.9354 |  | 2.562 |  |
| ASM 6m |  | 0.689 |  | 0.570 |  | 1.209 |  | | 0.227 |  | 1.993 |  | 0.6515 |  | 6.094 |  |
| New psychiatric symptoms 6m |  | 1.077 |  | 0.576 |  | 1.871 |  | | 0.061 |  | 2.935 |  | 0.9502 |  | 9.069 |  |
| New neurological symptoms 6m |  | 1.918 |  | 0.562 |  | 3.410 |  | | < .001 |  | 6.805 |  | 2.2603 |  | 20.490 |  |
|  | | | | | | | | | | | | | | | |  |
|  | | | | | | | | | | | | | | | |  |

**Table 5.** Multivariate logistic regression for the outcome of “long-term neurological symptoms” at 12 months. Note: estimates represent the log odds of "Long-term neurological symptoms 12-month = Yes" vs. "Long-term neurological symptoms 12-month = No". ASM: anti-seizure medications; DRE: drug-resistant epilepsy; FTB: focal-to-bilateral tonic-clonic seizures.
