# Supplementary material for: Exploring the long‐term effects of COVID‐19 in patients with epilepsy: A multicenter Italian observational study
Source: Epilepsia Open. 2025 Aug 9;10(5):1450–61. doi: 10.1002/epi4.70108 (PMC12514380; doi:10.1002/epi4.70108)
Supplement: Supplementary file 2 — Table S2 [file EPI4-10-1450-s002.docx]

|  | | | | | | | | | | | | | | | | |
| --- | --- | --- | --- | --- | --- | --- | --- | --- | --- | --- | --- | --- | --- | --- | --- | --- |
|  | | | | | | | | | | | | **95% Confidence Interval** | | | | |
| **Predictor** | | **Estimate** | | **SE** | | **Z** | | **p** | | **Odds ratio** | | **Lower** | | **Upper** | |  |
| Intercept |  | -3.392 |  | 0.866 |  | -3.916 |  | < .001 |  | 0.0336 |  | 0.00616 |  | 0.184 |  |  |
| Diagnosis: |  |  |  |  |  |  |  |  |  |  |  |  |  |  |  |  |
| Post Covid – Control |  | 0.741 |  | 0.630 |  | 1.176 |  | 0.240 |  | 2.0976 |  | 0.61002 |  | 7.213 |  |  |
| ASM baseline |  | 0.942 |  | 0.705 |  | 1.336 |  | 0.182 |  | 2.5654 |  | 0.64404 |  | 10.219 |  |  |
| DRE: |  |  |  |  |  |  |  |  |  |  |  |  |  |  |  |  |
| Yes – No |  | -1.932 |  | 1.494 |  | -1.293 |  | 0.196 |  | 0.1449 |  | 0.00775 |  | 2.710 |  |  |
| FTB frequency 6m |  | 0.226 |  | 0.385 |  | 0.588 |  | 0.557 |  | 1.2538 |  | 0.58987 |  | 2.665 |  |  |
| ASM 6m |  | -0.474 |  | 0.591 |  | -0.802 |  | 0.423 |  | 0.6224 |  | 0.19529 |  | 1.984 |  |  |
| New psychiatric symptoms 6m: |  |  |  |  |  |  |  |  |  |  |  |  |  |  |  |  |
| Yes – No |  | 3.097 |  | 0.619 |  | 5.002 |  | < .001 |  | 22.1311 |  | 6.57577 |  | 74.484 |  |  |
| New neurological symptoms 6m: |  |  |  |  |  |  |  |  |  |  |  |  |  |  |  |  |
| Yes – No |  | 1.530 |  | 0.646 |  | 2.368 |  | 0.018 |  | 4.6175 |  | 1.30157 |  | 16.382 |  |  |
|  | | | | | | | | | | | | | | | | |
|  | | | | | | | | | | | | | | | | |

**Table 6.** Multivariate logistic regression for the outcome “Long-term psychiatric symptoms” at 12-month. Note: estimates represent the log odds of "Long-term psychiatric symptoms 12-month = Yes" vs. "Long-term psychiatric symptoms 12-month = No". ASM: anti-seizure medications; DRE: drug-resistant epilepsy; FTB: focal-to-bilateral tonic-clonic seizures
